# Development and Evaluation of an Indirect ELISA for Strangles Based on an Untagged EQ8–SclF Fusion Protein

**DOI:** 10.3390/vetsci13090866

**Published:** 2026-08-26

**Authors:** Rongkuan Sun, Haoyu Zu, Min Wang, Wei Guo, Xiaojun Wang, Qian Yang

**Affiliations:** 1 College of Veterinary Medicine, Nanjing Agricultural University, Nanjing 210095, China; 18848113546@163.com; 2 State Key Laboratory for Animal Disease Control and Prevention, Harbin Veterinary Research Institute, Chinese Academy of Agricultural Sciences, Harbin 150069, China; 3 China-Kazakhstan Joint Laboratory for Herbivorous Animal Disease Research, Harbin Veterinary Research Institute, Chinese Academy of Agricultural Sciences, Harbin 150069, China; 4 Institute of Western Agriculture, Chinese Academy of Agricultural Sciences, Changji 831100, China

**Keywords:** strangles, *Streptococcus equi* subsp. *equi*, indirect ELISA, EQ8, SclF, diagnostic evaluations

## Abstract

Strangles, caused by *Streptococcus equi* subsp. *equi* (*S. equi*), is a highly contagious respiratory disease that causes substantial economic losses to the horse industry worldwide. Serological diagnosis currently relies mainly on the imported IDvet^®^ dual-antigen sandwich ELISA kit, which shows good diagnostic performance but is expensive and lacks a domestic alternative in China, thereby limiting its routine use in primary veterinary settings. The aim of this study was to develop a domestic indirect ELISA based on novel antigen targets and an untagged antigen purification strategy. Using comparative bioinformatics analyses, two conserved fragments with species-level specificity for *S. equi*, designated EQ8 (150 aa) and SclF (44 aa), were identified and fused for recombinant expression. The fusion protein was purified by GST affinity chromatography, cleaved with PreScission protease, and subjected to secondary purification to obtain an untagged EQ8–SclF protein. Western blotting and ELISA demonstrated that the purified protein retained good immunoreactivity and that the GST tag did not cause detectable cross-reactivity, with all OD_450_ values obtained using GST-coated plates remaining below 0.09, well under the cut-off value of 0.115. Checkerboard titration established the optimal assay conditions as an antigen coating concentration of 1 μg/mL and a serum dilution of 1:100. Using the IDvet kit as the reference method, 157 clinical serum samples (67 positive and 90 negative) were tested in parallel. The newly developed assay showed a relative sensitivity of 89.55% (60/67), a relative specificity of 94.44% (85/90), an overall agreement rate of 92.36% (145/157), and a kappa value of 0.84, indicating excellent agreement between the two methods. Good inter-assay reproducibility was observed, with coefficients of variation all below 9.29%. In conclusion, an indirect ELISA based on an untagged EQ8–SclF fusion protein was preliminarily established for the serological detection of strangles. This assay showed diagnostic performance highly consistent with that of the imported commercial kit while offering potential advantages in terms of lower cost, simpler operation, and elimination of tag-associated interference. It therefore shows potential as a domestic alternative for the serological diagnosis of strangles.

## Simple Summary

Strangles is a highly contagious respiratory disease in horses, caused by the bacterium *Streptococcus equi*. It spreads easily among horses and causes serious economic losses to the horse industry worldwide. Early and accurate detection of infected horses is essential for controlling outbreaks and preventing further spread. In this study, we developed a new blood test called an indirect ELISA to detect antibodies against the bacteria in horses. We used a specially designed protein fragment as the detection target, which was produced without any extra protein tags that might interfere with the test results. After testing 157 horse serum samples, we found that our new test gave results that were highly consistent with those of a commercial kit currently used as the reference method, while potentially being more affordable and easier to produce in China. This test provides a promising alternative for routine screening of strangles in horses, especially in local veterinary clinics, and may help horse owners and veterinarians take timely action to control disease spread.

## 1. Introduction

Strangles is a highly contagious respiratory disease caused by *Streptococcus equi* subsp. *equi* (*S. equi*), posing a major threat to equine health and causing substantial economic losses worldwide [1,2]. Disease control remains challenging for several reasons. The pathogen is widely distributed and exhibits strong environmental persistence [3]. In addition, approximately 10% of recovered horses become asymptomatic carriers, in which the organism can persist in the guttural pouch and be intermittently shed, thereby hampering eradication efforts [4,5]. Accordingly, serological methods capable of detecting antibodies induced by prior infection are of considerable value for surveillance and disease control programs.

Among currently available diagnostic approaches, real-time quantitative PCR (qPCR) is highly sensitive for pathogen detection but cannot distinguish viable from non-viable bacteria and depends heavily on appropriate sample collection, such as guttural pouch lavage, which limits its applicability in large-scale screening [6,7]. Enzyme-linked immunosorbent assay (ELISA), in contrast, is simple to perform and well suited to high-throughput screening, making it a widely used platform for serological surveillance [8]. However, its accuracy depends critically on antigen specificity. Indirect ELISAs based on the single SeM antigen have been reported to show cross-reactivity with the homologous SzM protein of *Streptococcus equi* subsp. *zooepidemicus* (*S. zooepidemicus*), resulting in a specificity of only approximately 77% and an undesirably high false-positive rate [1,9]. To address this limitation, Robinson et al. developed a dual-antigen indirect ELISA targeting antigen A (SEQ_2190) and antigen C (SeM), increasing the specificity to 99.3% [9,10]. Based on this principle, the French company IDvet developed the commercial IDvet^®^ dual-antigen sandwich ELISA kit for strangles, which has shown good diagnostic performance and is widely used as a reference method. Nevertheless, its dependence on importation and relatively high cost restrict its broader use in China, especially in primary veterinary settings. Therefore, development of a domestic alternative with comparable performance and lower cost is of practical significance.

Previous studies have shown that the eight antigens included in the Strangvac^®^ vaccine are conserved among global *S. equi* isolates [11]. Based on sequence comparisons, we identified a 150-amino-acid fragment from EQ8 (SEQ_0402) and a 44-amino-acid fragment from SclF (SEQ_0855). These two fragments are conserved in *S. equi* yet differ substantially from their homologous regions in *S. zooepidemicus*, suggesting species-level specificity. In the present study, the two fragments were fused and expressed as a GST-tagged recombinant protein, followed by PreScission protease cleavage to generate an untagged EQ8–SclF fusion antigen. An indirect ELISA was then established and optimized by checkerboard titration, and its diagnostic performance was evaluated using the IDvet kit as a reference. This work provides an experimental basis for the development of a domestic serological diagnostic tool for strangles.

## 2. Materials and Methods

### 2.1. Bacterial Strain and Serum Samples

The *S. equi* HLJ2018 strain was isolated and preserved by the Equine Infectious Diseases and Lentivirus Innovation Team at the State Key Laboratory for Animal Disease Control and Prevention, Harbin Veterinary Research Institute, Chinese Academy of Agricultural Sciences. These 157 serum samples were collected from horses in Harbin and surrounding areas of China, covering different age groups (including foals and adults), all from local farms without vaccination history, with sampling carried out between 2023 and 2024. A total of 157 serum samples used for method evaluation were classified using the IDvet^®^ Strangles dual-antigen sandwich ELISA kit (IDvet, Grabels, France), including 67 positive and 90 negative sera. Positive sera against equine infectious anemia virus (EIAV), equine herpesvirus (EHV), *Salmonella abortus equi*, equine arteritis virus (EAV), and equine influenza virus (EIV) were also provided by the same laboratory for specificity testing.

### 2.2. Screening of Species-Level Antigenic Fragments

Based on previous studies indicating that EQ8 (SEQ_0402) and SclF (SEQ_0855) have potential as *S. equi* vaccine candidate antigens, these proteins were selected as candidate diagnostic targets. Amino acid sequences encoded by EQ8 and SclF in *S. equi* strain 4047 (GenBank: FM204883.1) and *S. equi* ATCC 39506 (GenBank: CP021972.1) were compared with corresponding homologous sequences from *S. zooepidemicus* ATCC 35246 (GenBank: CP002904.1), *S. zooepidemicus* OH-71950 (GenBank: CP046040.1), and related strains using SnapGene (8.2.1) and Geneious Prime (2024.0.5). Conserved and diagnostically specific regions were then selected.

### 2.3. Construction of the Recombinant Plasmid

Based on the gene sequence of *S. equi* 4047 (GenBank: FM204883.1) and the principles of homologous recombination, three pairs of primers were designed in SnapGene for amplification of EQ8 (SEQ_0402) and SclF (SEQ_0855). Specifically, a *Bam*H I site and a homologous arm to pGEX-6p-1-R were introduced at the 5′ end of EQ8-F; a flexible linker (G_2_S)_4_ and a homologous arm to SclF-F were introduced at the 5′ end of EQ8-R; and an *Xho*I site and a homologous arm to pGEX-6p-1-F were introduced at the 5′ end of SclF-R (Supplementary Figure S1). Primers were synthesized (Saissons Biotechnology, Harbin, China). Primer sequences and amplicon sizes are listed in Table 1.

Using genomic DNA of *S. equi* HLJ2018 as a template, EQ8 and SclF fragments were amplified with primer pairs EQ8-F/EQ8-R and SclF-F/SclF-R, respectively. The pGEX-6p-1 vector backbone was linearized by PCR with pGEX-6p-1-F/pGEX-6p-1-R. The resulting PCR products were assembled using 2× EasyFusion Assembly Master Mix (NCMBiotech, Suzhou, China) at 50 °C for 30 min. The ligation product was transformed into *E. coli* DH5α competent cells (2nd Lab, Shanghai, China), plated on ampicillin-containing LB agar, and incubated at 37 °C. Single colonies were cultured at 37 °C and 180 rpm for 12 h and then sequenced (Kumei Biotechnology, Harbin, China). Positive recombinant plasmids were further verified by double digestion with *Bam*H I and *Xho*I (Thermo Fisher Scientific, Waltham, MA, USA), followed by 1% agarose gel electrophoresis and sequencing confirmation.

### 2.4. Expression and Purification of Recombinant Protein

The recombinant plasmid pGEX-6p-1-EQ8–SclF was transformed into *E. coli* BL21(DE3) competent cells (Beyotime, Shanghai, China) and plated on ampicillin-containing LB agar. After incubation at 37 °C for 12 h, a positive colony was inoculated into 5 mL LB broth containing ampicillin and cultured at 37 °C with shaking until the OD_600_ reached 0.6–0.8. Protein expression was induced with 0.5 mM IPTG at 16 °C and 120 rpm for 24 h. Cells were harvested by centrifugation, disrupted by ultrasonication, and the supernatant was subjected to GST-tag protein purification using a commercial GST purification kit to obtain the GST-EQ8–SclF fusion protein.

### 2.5. Preparation of Untagged EQ8–SclF Protein

Purified GST-EQ8–SclF fusion protein (1 mg/mL) was mixed with PreScission protease (1 mg/mL; Beyotime, Shanghai, China) and incubated at 4 °C for 16 h with rotation. After enzymatic cleavage, the reaction mixture was incubated with Glutathione Resin (Beyotime, Shanghai, China) at 4 °C for 1 h to adsorb and remove the GST tag, GST-tagged PreScission protease, and uncleaved fusion protein. The flow-through containing the target protein was collected. Samples were mixed with 5 × SDS loading buffer, boiled at 100 °C for 10 min, and analyzed by SDS-PAGE using precast protein gels (ACE, Beijing, China). Protein concentration was determined using the Pierce™ BCA Protein Assay Kit (Thermo Fisher Scientific, Waltham, MA, USA). Purified protein was aliquoted and stored at −80 °C until use.

### 2.6. Western Blot Analysis

Protein samples included purified GST-EQ8–SclF fusion protein, the PreScission protease cleavage mixture, purified untagged EQ8–SclF protein, and GST protein (SinoBiological, Beijing, China) as a control. After SDS-PAGE, proteins were transferred onto nitrocellulose membranes. Membranes were blocked with 5% skim milk (Biosharp, Beijing, China) for 2 h at room temperature with gentle shaking (60 rpm), followed by three washes with 1× TBST (Merck-Millipore, Burlington, MA, USA), 10 min each. Membranes were then incubated overnight at 4 °C with either strangles-positive equine serum diluted 1:500 in 5% skim milk or rabbit polyclonal anti-GST antibody (Proteintech, Rosemont, IL, USA) diluted 1:5000 in 5% skim milk. After three washes with TBST, membranes were incubated for 2 h at room temperature with either fluorescent rabbit anti-horse IgG (H + L) secondary antibody (NOVUS, Centennial, CO, USA; 1:10,000) or fluorescent goat anti-rabbit IgG secondary antibody (Biosciences, Lincoln, NE, USA; 1:10,000). Following three additional washes, membranes were scanned using an ODYSSEY CLx infrared imaging system.

### 2.7. Establishment and Optimization of the Indirect ELISA

Purified untagged EQ8–SclF protein was used as the coating antigen, *S. equi*-positive and -negative sera served as primary antibodies, and HRP-conjugated goat anti-horse IgG (KPL, Gaithersburg, MD, USA) was used as the secondary antibody. Briefly, the antigen was diluted in 0.05 mol/L carbonate buffer (pH 9.6), added at 100 μL/well, and incubated at 4 °C for 12 h. Plates were washed three times with PBST and blocked with 200 μL/well of 5% skim milk at 37 °C for 1.5 h. Serum samples diluted in 5% BSA (Solarbio, Beijing, China) were then added at 100 μL/well and incubated at 37 °C for 1 h. Subsequently, HRP-conjugated goat anti-horse IgG diluted in 5% BSA at 1:10,000 was added at 100 μL/well and incubated at 37 °C for 1 h. Color was developed with 100 μL/well TMB substrate (InnoReagents, Huzhou, China) for 10 min at room temperature in the dark, and the reaction was stopped with 50 μL/well of 2 mol/L H_2_SO_4_. OD_450_ values were then measured.

A checkerboard titration was performed to optimize the antigen coating concentration and serum dilution. Antigen concentrations of 0.5, 1, 2, 5, 10, and 20 μg/mL were tested, and positive sera were assayed at dilutions of 1:50, 1:100, 1:200, 1:400, 1:500, 1:600, 1:800, and 1:1000. Negative sera were diluted in parallel. The combination yielding the maximum positive-to-negative (P/N) ratio was considered optimal. Based on the selected antigen concentration and serum dilution, additional parameters, including coating condition, blocking solution, serum diluent, secondary antibody diluent, secondary antibody working dilution, and TMB development time, were further optimized.

### 2.8. Determination of the Cut-Off Value

Eighteen known *S. equi*-negative sera, collected from newborn foals (approximately 3–4 months old) and confirmed as negative by the IDvet kit, were tested using the established indirect ELISA, with each sample run in duplicate. The mean OD_450_ value (X) and standard deviation (SD) of the negative sera were calculated. According to the criterion of X + 3SD, samples with OD_450_ values above this threshold were considered positive, corresponding to a confidence level of 99.9%.

### 2.9. Specificity Testing

To assess analytical specificity, positive sera against EIAV, EHV, *S. abortus equi*, EAV, and EIV were tested at a 1:100 dilution using the established indirect ELISA. Each group was tested in duplicate, and *S. equi*-positive and -negative sera were included as controls. In addition, GST protein (1 μg/mL) was used as a coating antigen to test 20 *S. equi*-positive sera identified by the IDvet kit, in order to evaluate whether the GST tag produced any cross-reactive interference.

### 2.10. Inter-Assay Reproducibility

Twenty-four known *S. equi* serum samples covering different antibody levels were tested on two independently coated ELISA plates, with each sample run in duplicate. Coefficients of variation (CVs) were calculated as SD/mean × 100%.

### 2.11. Comparison with the Commercial Kit and Statistical Analysis

The newly developed indirect ELISA and the IDvet kit (IDvet, Grabels, France) were used in parallel to test 157 clinical serum samples.

All data were analyzed using GraphPad Prism 9.0. Shapiro–Wilk normality and Levene’s homogeneity tests were performed before one-way ANOVA where applicable. Data are presented as mean ± SD. One-way ANOVA followed by Tukey’s HSD test was used to evaluate OD value differences between different batches in the inter-assay reproducibility test. Statistical significance was denoted as ns (*p* ≥ 0.05), * (*p* < 0.05), ** (*p* < 0.01), and *** (*p* < 0.001). The 95% confidence intervals for sensitivity and specificity were calculated using the Wilson score method. The confidence interval for the Kappa value was calculated using the standard error method.

## 3. Results

### 3.1. Identification of Species-Level Antigenic Fragments

**Figure 1:**
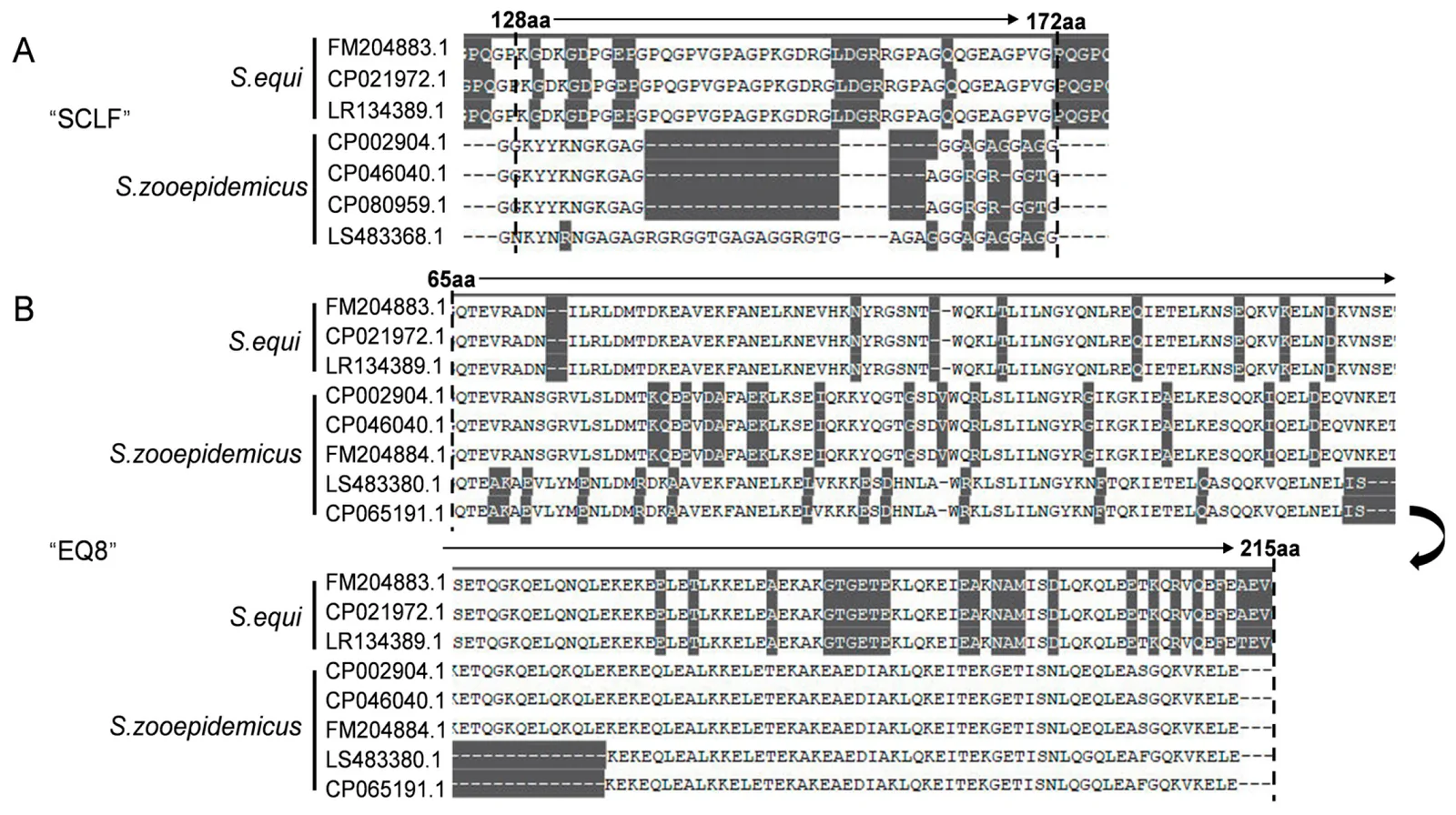
Amino acid sequence comparison of the selected antigenic fragments. (**A**) Sequence alignment of the SclF fragment between *S. equi* and *S. zooepidemicus*, showing that the corresponding region is absent in *S. zooepidemicus*. (**B**) Sequence alignment of the EQ8 fragment between *S. equi* and *S. zooepidemicus*, showing low sequence homology.

Through sequence comparison, a 150-amino-acid fragment from EQ8 (SEQ_0402) and a 44-amino-acid fragment from SclF (SEQ_0855) were selected and designated “EQ8” and “SclF”, respectively. Both fragments were conserved and specific in *S. equi* subsp. *equi* reference strains, including strain 4047 (GenBank: FM204883.1) and ATCC 39506 (GenBank: CP021972.1). The SclF fragment was absent from the corresponding region in *S. equi* subsp. *zooepidemicus* reference strains, including ATCC 35246 (GenBank: CP002904.1) and OH-71950 (GenBank: CP046040.1) (Figure 1A). For EQ8, the N-terminal region showed poor conservation in *S. zooepidemicus*, whereas the C-terminal region was relatively more conserved but still differed substantially from the *S. equi* sequence, with overall low homology (Figure 1B). We successfully identified the EQ8 (150 aa) and SclF (44 aa) fragments. Both fragments possess good species-level specificity for *S. equi* and exhibit low homology with the corresponding homologous sequences of *S. zooepidemicus*, making them promising molecular targets for the development of a specific diagnostic reagent.

### 3.2. Construction and Verification of the Recombinant Plasmid

**Figure 2:**
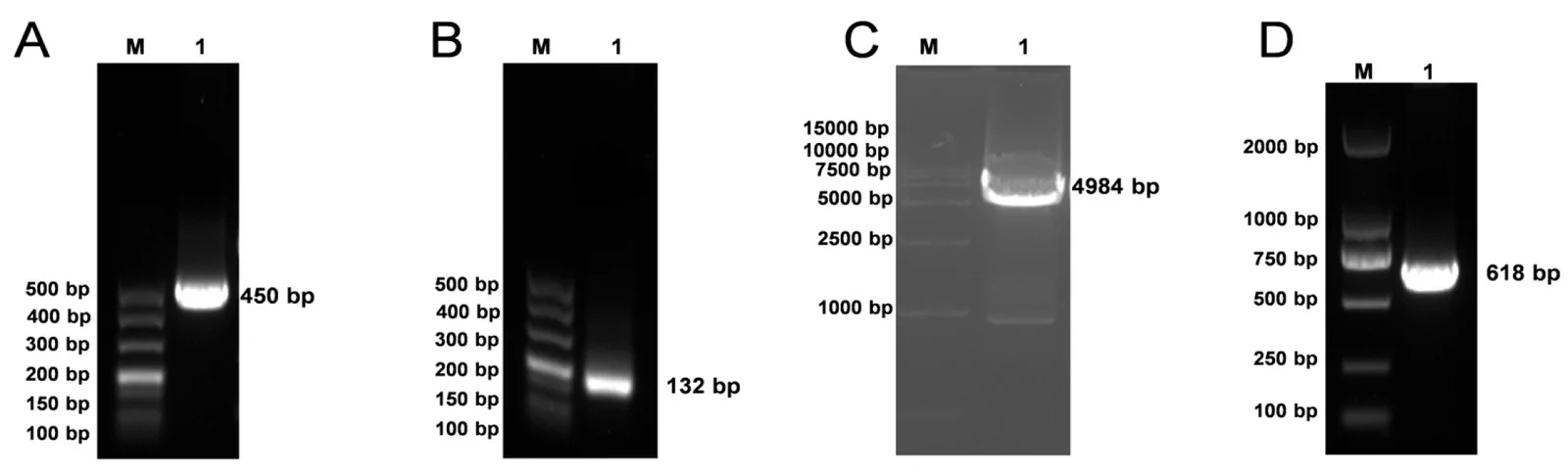
PCR amplification and restriction digestion verification of the recombinant plasmid pGEX-6p-1-EQ8–SclF. (**A**) PCR amplification of the EQ8 fragment. Lane M: DL500 DNA marker; Lane 1: EQ8 amplicon (450 bp). (**B**) PCR amplification of the SclF fragment. Lane M: DL500 DNA marker; Lane 1: SclF amplicon (132 bp). (**C**) Linearized pGEX-6p-1 vector fragment. Lane M: DL15000 DNA marker; Lane 1: linearized pGEX-6p-1 vector (4984 bp). (**D**) Double digestion verification of the recombinant plasmid with BamH I and XhoI. Lane M: DL2000 DNA marker; Lane 1: released EQ8–SclF insert fragment (618 bp). The expected sizes of the amplified and digested fragments were consistent with the design, confirming successful construction of the recombinant plasmid.

To express the EQ8–SclF fusion protein, the recombinant plasmid pGEX-6p-1-EQ8–SclF was constructed. Using genomic DNA from *S. equi* HLJ2018 as the template, PCR amplification yielded fragments of the expected sizes for EQ8 (450 bp) and SclF (132 bp) (Figure 2A,B). These fragments were assembled with the linearized pGEX-6p-1 vector (Figure 2C) by homologous recombination. Double digestion with *Bam*H I and *Xho*I released an approximately 618 bp insert (Figure 2D), which was fully consistent with the expected size of the EQ8-linker-SclF fusion gene (EQ8, 450 bp; SclF, 132 bp; flexible linker, 36 bp). Sequencing further confirmed that the insert sequence was correct, demonstrating successful construction of the recombinant plasmid.

### 3.3. Expression, Purification, and Identification of the Recombinant Protein

**Figure 3:**
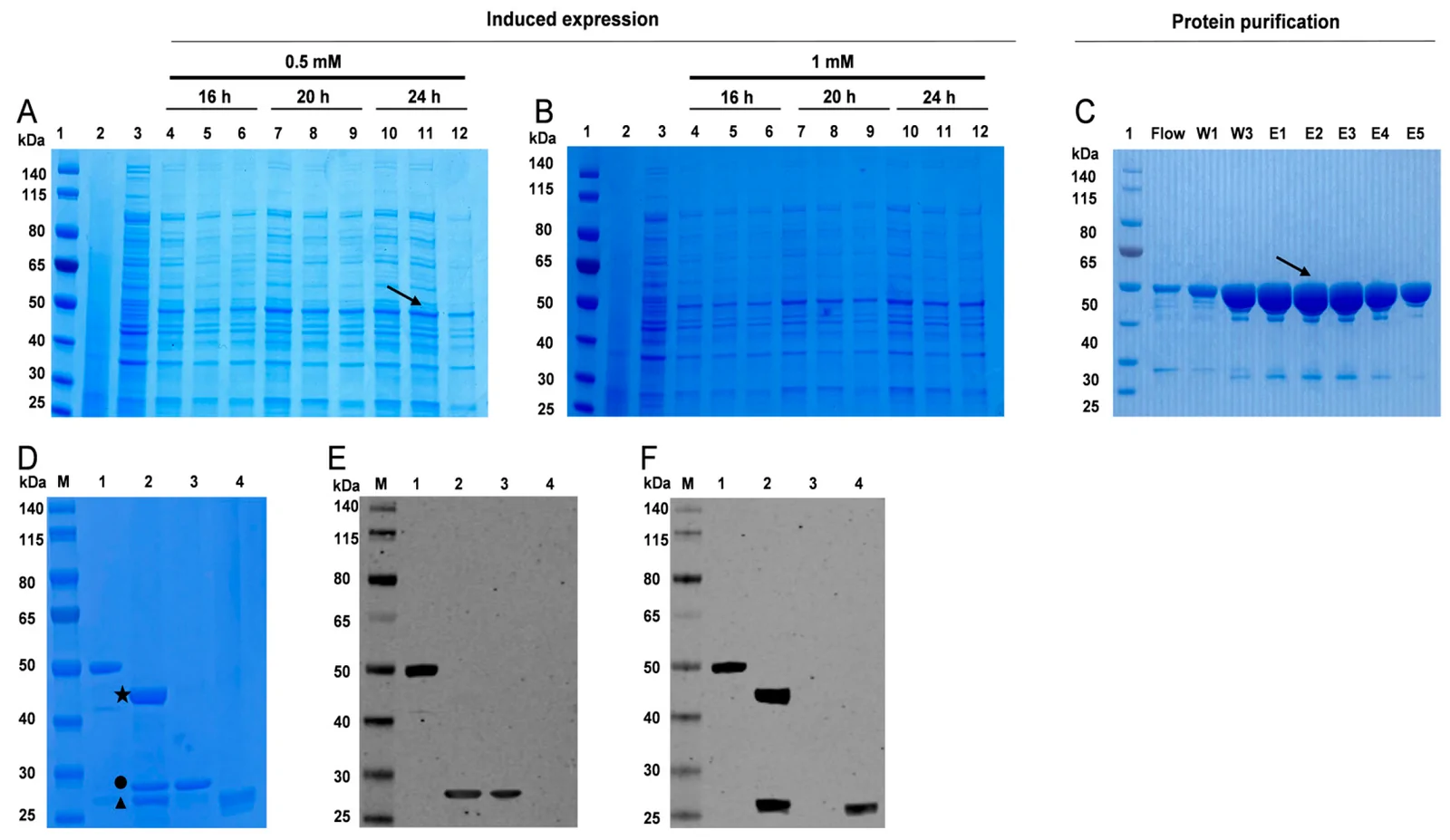
Expression, purification, and Western blot verification of the GST-EQ8–SclF fusion protein. (**A**) SDS-PAGE analysis of GST-EQ8–SclF fusion protein expression induced with 0.5 mM IPTG at 16 °C. Lanes 1–12: protein marker; empty vector control; uninduced cells; induced total bacteria (16 h); induced supernatant (16 h); induced pellet (16 h); induced total bacteria (20 h); induced supernatant (20 h); induced pellet (20 h); induced total bacteria (24 h); induced supernatant (24 h); induced pellet (24 h). The arrow indicates the GST-EQ8–SclF fusion protein band expressed under the optimal induction conditions. (**B**) SDS-PAGE analysis of GST-EQ8–SclF fusion protein expression induced with 1 mM IPTG at 16 °C. Lanes 1–12 are arranged as in panel (**A**). (**C**) Purification profile of GST-EQ8–SclF by glutathione affinity chromatography. Flow, W1, W3, E1–E5 represent flow-through, wash fraction 1, wash fraction 3, and elution fractions 1–5, respectively. The arrow indicates the target protein in the elution fractions. (**D**) SDS-PAGE analysis of purified and cleaved proteins. Lane M: protein marker; Lane 1: purified GST-EQ8–SclF fusion protein; Lane 2: cleavage mixture after PreScission protease digestion (★: GST-tagged PreScission protease; ●: untagged EQ8–SclF; ▲: released GST tag); Lane 3: purified untagged EQ8–SclF protein after secondary purification; Lane 4: purified GST tag protein. (**E**) Western blot probed with strangles-positive equine serum. Lanes M and 1–4 are as described in panel (**D**). (**F**) Western blot probed with anti-GST antibody. Lanes M and 1–4 are as described in panel (**D**).

Under the optimized induction conditions (0.5 mM IPTG, 16 °C, 120 rpm, 24 h), SDS-PAGE showed a specific band at approximately 50 kDa, consistent with the predicted molecular weight of GST-EQ8–SclF (Figure 3A,B). The soluble fraction was purified by glutathione affinity chromatography to yield a high-purity product (Figure 3C). After cleavage with PreScission protease at 4 °C for 16 h, the 50 kDa band disappeared and new bands appeared at approximately 28–30 kDa, approximately 44 kDa (PreScission protease), and approximately 26 kDa (released GST tag) (Figure 3D, lane 2). Notably, the apparent molecular weight of EQ8–SclF on SDS-PAGE (approximately 28–30 kDa) was significantly higher than its theoretical value (approximately 24 kDa) and also higher than that of the released GST tag (~26 kDa). However, this migration shift did not affect the antigen’s immunoreactivity (Figure 3E). Subsequent adsorption with glutathione resin removed GST-containing contaminants, yielding highly purified untagged EQ8–SclF protein (Figure 3D, lane 3).

Western blotting further confirmed the immunoreactivity of the purified protein. When strangles-positive equine serum was used as the primary antibody, strong specific signals were detected for the uncleaved fusion protein (lane 1) and for the target protein band in lanes 2 and 3 (28–30 kDa), whereas no signal was observed for the released GST tag (~26 kDa in lane 2) or the GST control protein (lane 4) (Figure 3E). This indicated that antibodies in the positive equine serum specifically recognized antigenic epitopes within EQ8 and/or SclF and that the cleaved target protein retained strong immunoreactivity. Consistently, anti-GST antibody reacted only with GST-containing components, including the uncleaved fusion protein (lane 1), GST-tagged cleavage products in lane 2, and GST control protein (lane 4), but not with the target protein in lanes 2 and 3 (Figure 3F). This confirmed that the final purified product contained no GST tag. Together, these results demonstrate that a highly purified, GST-free, and immunoreactive EQ8–SclF protein was successfully prepared for subsequent ELISA development.

### 3.4. Optimized Conditions for the Indirect ELISA and Its Diagnostic Performance

**Figure 4:**
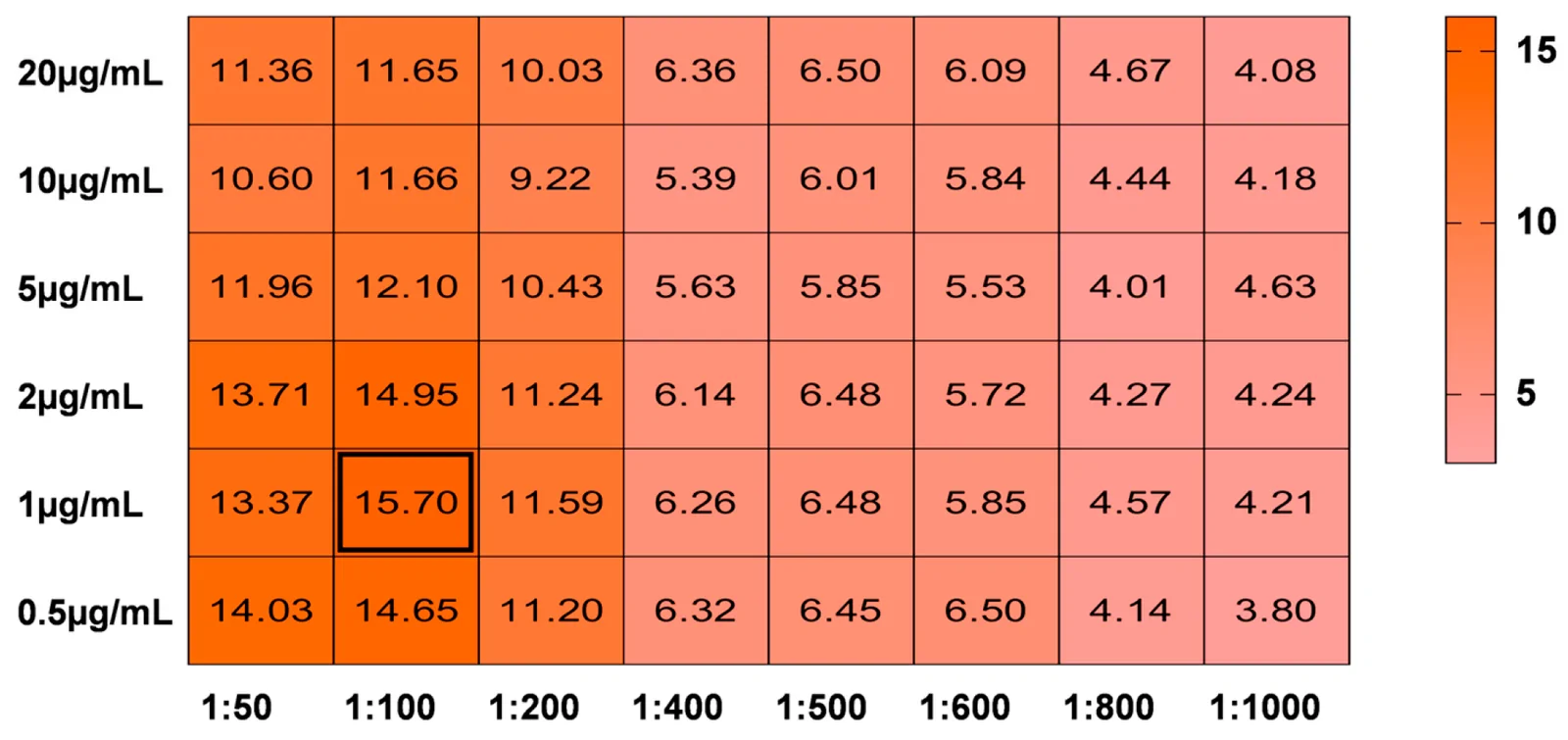
Checkerboard titration for optimization of antigen coating concentration and serum dilution. Each square in the heatmap represents one combination of antigen coating concentration and serum dilution, with the positive-to-negative (P/N) OD_450_ ratio shown in each cell. The color gradient ranges from light red (low P/N ratio) to dark red (high P/N ratio). The highest P/N ratio (15.7) was obtained at an antigen coating concentration of 1 μg/mL and a serum dilution of 1:100, which was therefore selected as the optimal condition and is highlighted with a box in the figure.

Checkerboard titration was used to determine the optimal antigen coating concentration and serum dilution. The highest P/N ratio (15.7) was obtained when the antigen coating concentration was 1 μg/mL and the serum dilution was 1:100; these conditions were therefore selected as optimal for the assay (Figure 4). Other optimized reaction conditions are summarized in Table 2.

### 3.5. Performance Evaluation of the Indirect ELISA

Using 18 *S. equi*-negative sera tested in duplicate, the mean OD_450_ value (X) and standard deviation (SD) were calculated, and the cut-off value was defined as X + 3SD. The resulting values were X = 0.08128 and SD = 0.01142, yielding a cut-off value of 0.11554. Samples with OD_450_ values above this threshold were considered positive, corresponding to a confidence level of 99.9%.

**Figure 5:**
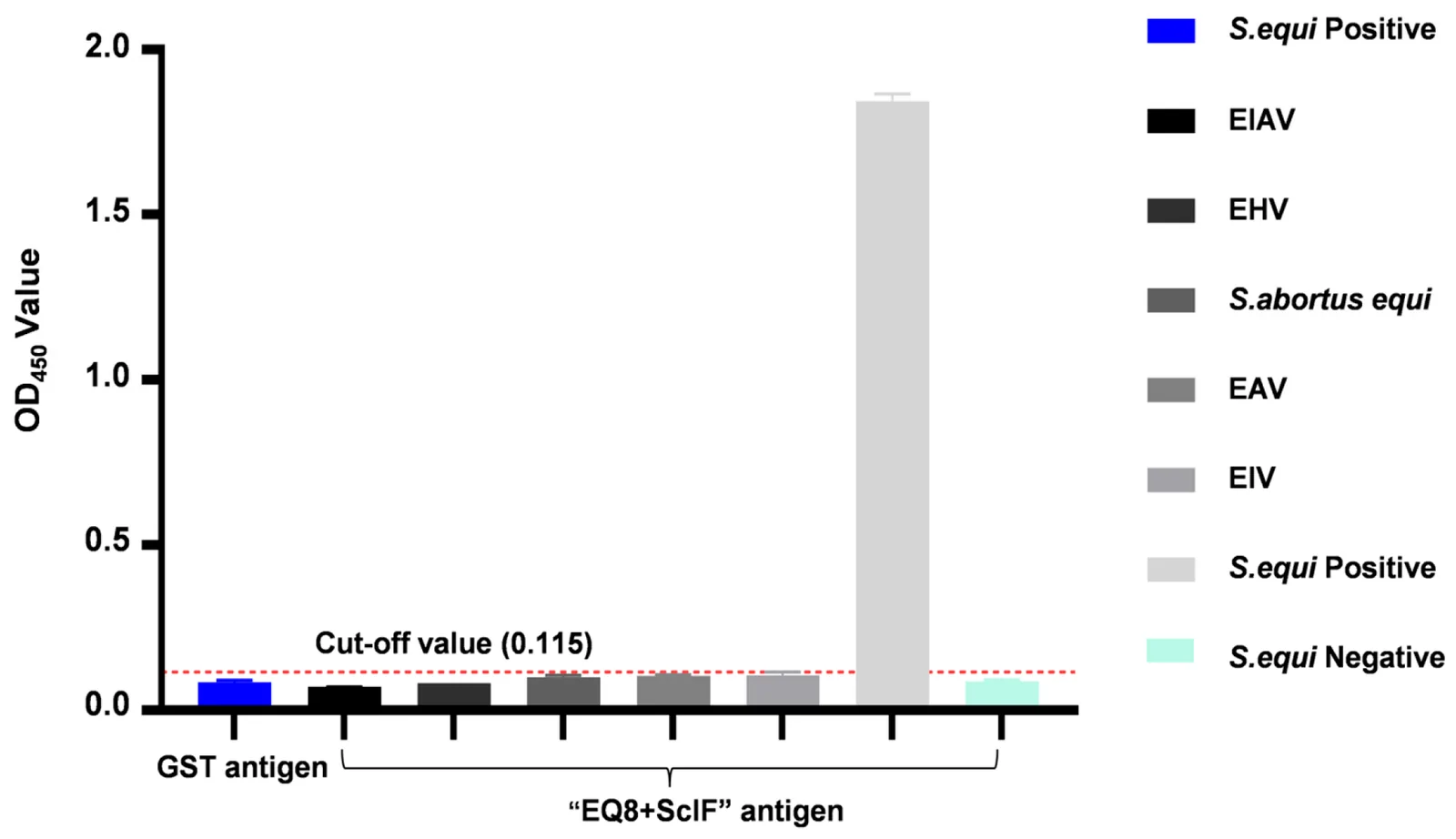
Specificity analysis of the established indirect ELISA.GST-coated antigen (blue bars) was tested against *S. equi*-positive sera (n = 20), and EQ8–SclF-coated antigen (gray bars) was tested against positive sera for EIAV, EHV, *S. abortus equi*, EAV, and EIV, as well as *S. equi*-positive and -negative sera. Data are presented as mean OD_450_ ± SD. The dashed line indicates the assay cut-off value (0.115).

Specificity testing showed that positive sera against EIAV, EHV, *S. abortus equi*, EAV, and EIV all yielded negative results in the established ELISA. In addition, when GST protein was used as the coating antigen to test 20 *S. equi*-positive sera identified by the IDvet kit, OD_450_ values ranged from 0.055 to 0.089 (mean ± SD: 0.0793 ± 0.0079), all below the cut-off value of 0.11554. This indicates that the GST tag did not cause detectable cross-reactivity, in agreement with the Western blot results. Together, these findings suggest that the assay has promising analytical specificity for *S. equi* infection and further support the absence of GST-derived interference (Figure 5).

Inter-assay reproducibility testing showed that the CVs of the 24 serum samples ranged from 0.05% to 9.29%, all below 10%, indicating good reproducibility of the established method. Representative results for four samples are shown in Table 3. The complete data for all 24 samples are provided in (Supplementary Table S1).

**Table 3:** Inter-assay reproducibility of the indirect ELISA.

Serum Sample	Status	Mean OD_450_ ± SD	Inter-Assay CV (%)
Sample A	Negative	0.080 ± 0.004	5.30
Sample B	Negative	0.083 ± 0.001	1.69
Sample C	Weakly Positive	0.220 ± 0.009	4.18
Sample D	Strongly Positive	1.654 ± 0.047	2.82

### 3.6. Comparison with the Commercial IDvet Kit

The newly developed indirect ELISA and the IDvet kit were used in parallel to test 157 clinical serum samples. Compared with the IDvet kit, the new ELISA showed an overall agreement rate of 92.36% (145/157) and a kappa value of 0.84, indicating excellent agreement. The relative sensitivity was 89.55% (95% CI: 80.6–95.7%), and the relative specificity was 94.44% (95% CI: 88.3–98.3%) (Table 4).

**Figure 6:**
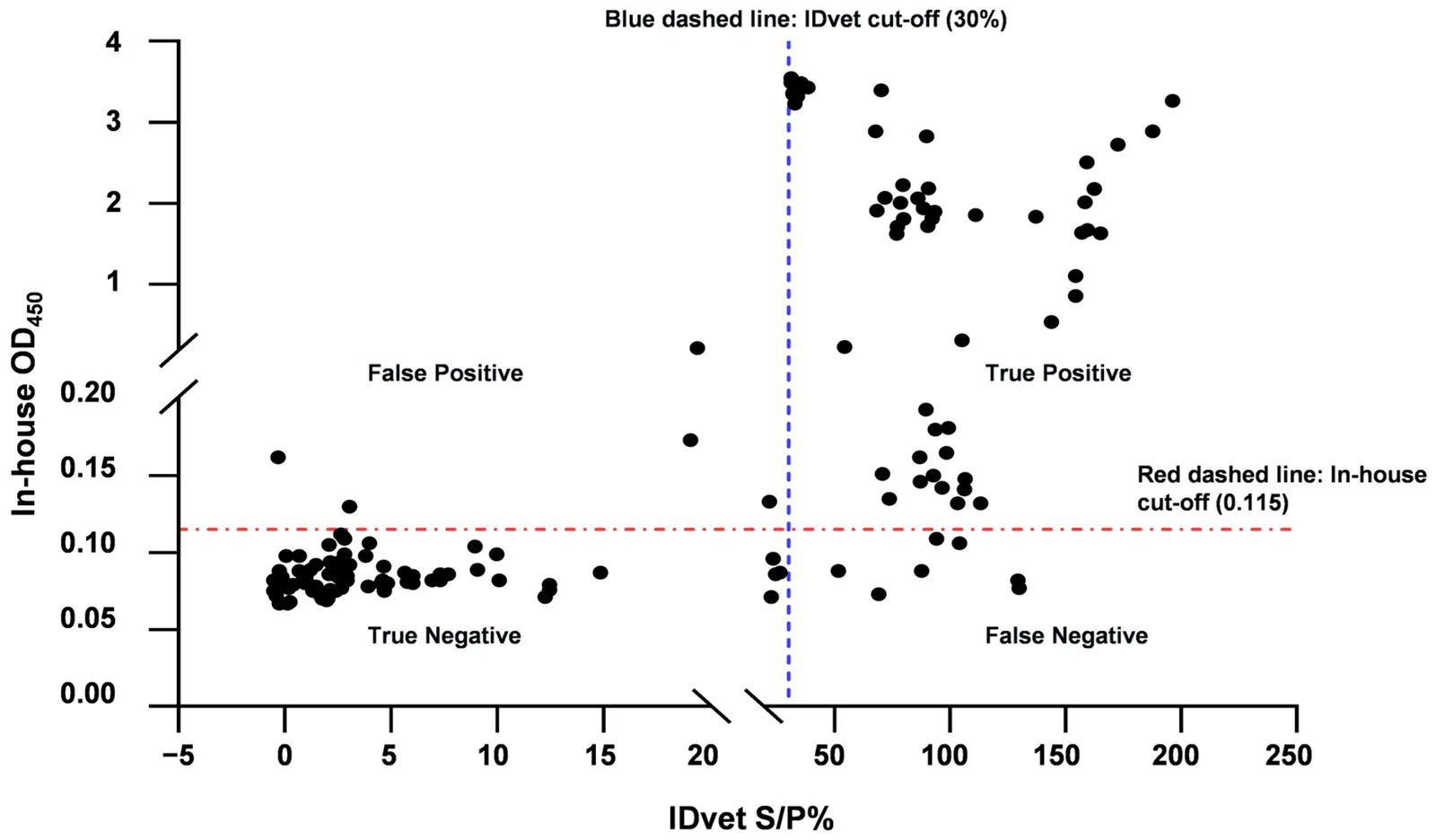
Agreement scatter plot between the newly developed ELISA and the IDvet kit. Scatter plot of OD_450_ values obtained with the new ELISA versus S/P% values obtained with the IDvet kit for 157 clinical serum samples. The blue dashed line indicates the IDvet cut-off (S/P% = 30; samples with S/P% ≥ 30 were considered positive), and the red dashed line indicates the cut-off of the new ELISA (OD = 0.115; samples with OD > 0.115 were considered positive). Based on these thresholds, samples were distributed into four quadrants: true negative (lower left), false positive (upper left), false negative (lower right), and true positive (upper right).

A consistency scatter plot showed that most samples clustered in the true-negative (lower left) and true-positive (upper right) quadrants, whereas only a small number of discrepant samples were distributed in the false-positive (upper left) and false-negative (lower right) quadrants (Figure 6). These results indicate that the newly established ELISA has diagnostic performance highly consistent with that of the commercial reference kit.

## 4. Discussion

The central challenge in serological diagnosis of strangles is to achieve broad accessibility and practical applicability without compromising diagnostic specificity. At present, the IDvet kit, which is based on the SeM and SEQ_2190 antigens, is widely recognized as a reference serological method and has shown stable diagnostic performance. However, its relatively high cost and dependence on importation limit its use in primary veterinary settings. In addition, earlier ELISA methods based on the single SeM antigen suffered from inadequate specificity because of cross-reactivity with the closely related *S. zooepidemicus*, making them insufficient for disease control purposes. Therefore, it is both necessary and practical to develop a domestic diagnostic strategy with improved affordability and good specificity.

To this end, the present study did not follow the antigen design used in existing diagnostic systems, such as SeM or SEQ_2190, but instead pursued an alternative route based on two key considerations: selection of highly specific diagnostic antigens and optimization of recombinant protein preparation. The EQ8 and SclF fragments, which are specific to *S. equi*, were selected as diagnostic targets. Sequence comparison showed that the SclF fragment is absent from *S. zooepidemicus*, while the EQ8 fragment shares low homology with *S. zooepidemicus*. Both fragments are conserved among *S. equi* strains, which should help minimize cross-reactivity with related organisms. In addition, a GST-fusion expression system followed by enzymatic tag removal was used to generate an untagged antigen, thereby eliminating potential nonspecific background associated with the purification tag and improving assay specificity.

Using the IDvet kit as a reference, the newly established ELISA achieved an overall agreement of 92.36% and a kappa value of 0.84, indicating excellent agreement between the two methods [12]. The assay also showed a relative specificity of 94.44% and good inter-assay reproducibility (CV < 10%), supporting the feasibility of this technical strategy. These advantages may be attributable to three closely related factors. First, bioinformatic screening supported species-level specificity of the selected antigenic fragments, reducing the risk of cross-reactivity at the source. Second, the untagged purification strategy was validated by Western blotting, which confirmed complete removal of the GST tag and absence of tag-derived interference, supporting that assay specificity was primarily determined by the immunoreactive properties of the EQ8–SclF antigen itself. Third, fusion of EQ8 and SclF into a single recombinant protein ensured a fixed antigen ratio, which is advantageous for standardized reagent production and may also facilitate more complete epitope presentation and improved antibody capture efficiency [13,14]. We acknowledge that experimental validation using *S. zooepidemicus*-positive sera was not performed in this study, which represents an important limitation. Although bioinformatic analysis indicated that the SclF fragment is absent from the corresponding genomic region of *S. zooepidemicus* and that EQ8 shares only low homology with the corresponding sequences in *S. zooepidemicus*, these sequence-based findings cannot replace serological validation. Future studies will address this gap as soon as such samples become available.

Interestingly, the purified untagged EQ8–SclF protein migrated at approximately 28–30 kDa on SDS-PAGE, which was higher than the predicted molecular weight of approximately 24 kDa. This discrepancy may be explained by anomalous electrophoretic migration associated with the acidic nature of the protein (predicted pI 4.91). Acidic domains may reduce SDS binding because of electrostatic effects, thereby slowing electrophoretic mobility [15,16,17]. Importantly, this migration shift did not affect immunoreactivity, suggesting that the overall antigenic conformation remained intact and functionally suitable for diagnostic use [18].

A small number of discrepant results between the newly developed ELISA and the IDvet kit may also provide useful insights into the serological response to *S. equi* infection. We hypothesize that the seven samples that were negative by the new ELISA but positive by the IDvet kit suggest that, in some animals, antibody responses against SeM or SEQ_2190 may predominate, as SeM is known to induce strong antibody responses after natural infection [19,20]. By contrast, EQ8 and SclF are conserved antigens whose antibody kinetics may differ from those of SeM. Conversely, we speculate that the five samples that were positive by the new ELISA but negative by the IDvet kit may represent animals in late infection or recovery, in which antibodies against SeM or SEQ_2190 had waned while antibodies against EQ8 or SclF persisted longer [21]. However, these explanations are speculative and require further experimental validation.

It should be noted that the present diagnostic evaluation was conducted using the commercial IDvet kit as the reference method. Although this kit is widely used, it is not a pathogen-based gold standard such as qPCR or bacterial culture [22,23]. Therefore, the sensitivity and specificity values reported here should be interpreted as relative performance measures against the reference kit rather than absolute measures against confirmed infection status. Future studies should incorporate sera from animals classified by qPCR and/or bacterial culture to more rigorously define the diagnostic performance of this assay relative to etiological gold standards. In addition, although no GST-associated cross-reactivity was detected in the present sample set, the sample size for this analysis was limited. Nevertheless, the untagged antigen preparation strategy used here inherently minimizes the risk of tag-related nonspecific interference. We acknowledge that the cut-off value was determined using only 18 negative sera, which is a relatively small sample size. Future studies with a larger panel of negative sera are needed to further optimize the cut-off. In addition, we acknowledge that intra-assay reproducibility was not evaluated in the present study. Future studies will include intra-assay reproducibility assessments to more comprehensively characterize the stability and repeatability of the assay.

Compared with the imported IDvet kit, all reagents used in the present assay can be sourced domestically, potentially reducing cost and improving accessibility for large-scale serological screening in primary veterinary settings, although a formal cost analysis was not performed in this study. From a strategic perspective, this method may also have potential for future expansion. The currently licensed Strangvac^®^ subunit vaccine uses a multicomponent antigen design [11], whereas the corresponding diagnostic kit (IDvet^®^) relies on SeM and SEQ_2190 [9]. Importantly, the EQ8 and SclF targets used in the present study are distinct from those used in these systems. In particular, the 44-aa SclF-specific fragment selected here lies in a different sequence region from the SclF-derived fragment included in Strangvac^®^. This raises the possibility that horses immunized with Strangvac^®^ may not produce antibodies against the 44-aa diagnostic fragment used here, whereas naturally infected horses may do so, potentially conferring a DIVA-like (differentiating infected from vaccinated animals) characteristic. Although this possibility remains theoretical at present and requires validation using sera from vaccinated horses, it may provide a useful basis for future vaccine monitoring and epidemiological studies should such vaccines become available in China.

In summary, this study established a domestic indirect ELISA for strangles with diagnostic performance highly consistent with that of an imported commercial kit. Nevertheless, although the assay was validated using 157 clinical serum samples, its potential applications in early infection, longitudinal disease monitoring, and characterization of antibody dynamics during recovery still require further evaluation using serial serum samples collected over time.

## 5. Conclusions

In this study, a new indirect ELISA for strangles was preliminarily established through species-level antigen screening and preparation of an untagged fusion protein. The assay showed high agreement with the commercial IDvet kit, with an overall agreement rate of 92.36%, a kappa value of 0.84, a relative sensitivity of 89.55%, a relative specificity of 94.44%, and good inter-assay reproducibility (CV < 10%). Moreover, this work validated an integrated technical workflow from antigen selection to protein preparation that differs from conventional antigen systems used in strangles serodiagnosis; thereby, it may serve as a potential technical reference for the development of domestic diagnostic reagents.

## Figures and Tables

**Table 1:** Primers used for PCR amplification.

Primer Name	Primer Sequence (5′→3′)	Amplicon Size (bp)
EQ8-F	aggggcccctgggatccCAAACAGAAGTACGGGCTGATAATATC	450 bp
EQ8-R	GCTTCCGCCGCTTCCGCCGCTTCCGCCGCTTCCGCCTACTTCAGCTTCAAACTCTTGAACC	
SclF-F	GGCGGAAGCGGCGGAAGCGGCGGAAGCGGCGGAAGCAAGGGTGATAAAGGAGACCCCGGA	132 bp
SclF-R	ctcgagtcaACCAACTGGGCCGGCTTCCCCTTGCT	
pGEX-6p-1-F	AGCCGGCCCAGTTGGTtgactcgagcggccgcatcgtg	4984 bp
pGEX-6p-1-R	atcccaggggcccctggaacagaacttccagatccg	

**Table 2:** Summary of optimal reaction conditions for the indirect ELISA.

Parameter	Tested Range	Optimal Condition
Antigen coating concentration (µg/mL)	20, 10, 5, 2, 1, 0.5	1
Serum dilution	1:50, 1:100, 1:200, 1:400, 1:500, 1:600, 1:800, 1:1000	1:100
Coating condition	4 °C 12 h, 37 °C 2 h, 25 °C 2 h	4 °C 12 h
Blocking solution	Microplate stabilizers (type I, type II), 5% FBS, 5% BSA, 5% skim milk	5% FBS
Serum diluent	Commercial protein stabilizers (type I, type II), 5% FBS, 5% BSA, 5% skim milk	5% BSA
HRP-conjugated secondary antibody diluent	Commercial HRP conjugate stabilizers (types I, II, and III), 5% BSA, 5% skim milk	Commercial HRP conjugate stabilizer type I
Dilution of HRP-conjugated secondary antibody	1:1000, 1:2000, 1:5000, 1:10,000, 1:20,000	1:10,000
TMB color development time	6, 8, 10, 12 min	10 min

**Table 4:** Diagnostic performance of the newly developed ELISA compared with the commercial IDvet kit.

	IDvet Kit (Positive Sera)	IDvet Kit (Negative Sera)	Total Sera
iELISA (Positive sera)	60	5	65
iELISA (Negative sera)	7	85	92
Total sera	67	90	157
Performance parameters			
Relative Sensitivity	89.55%		
Relative Specificity		94.44%	
Overall agreement rate			92.36%
Kappa value			0.84

**Note:** Relative sensitivity, relative specificity, overall agreement rate, and Kappa value were calculated using the IDvet kit results as the reference standard.

## Data Availability

The original contributions presented in this study are included in the article/Supplementary Materials. Further inquiries can be directed to the corresponding authors.

## Supplementary Materials

The following supporting information can be downloaded at: https://www.mdpi.com/article/10.3390/vetsci13090866/s1, Figure S1. SDS-PAGE analysis of purified and cleaved proteins; Figure S2. Western blot probed with strangles-positive equine serum; Figure S3. Western blot probed with anti-GST antibody; Figure S4. PCR amplification and restriction digestion verification of the recombinant plasmid pGEX-6p-1-EQ8–SclF; Table S1. Complete data for inter-assay reproducibility of the indirect ELISA.
