# Supplementary material for: Development and Evaluation of an Indirect ELISA for Strangles Based on an Untagged EQ8–SclF Fusion Protein
Source: Vet Sci. 2026 Aug 26;13(9):866. doi: 10.3390/vetsci13090866 (PMC13611558; doi:10.3390/vetsci13090866)
Supplement: Supplementary file 1 [file vetsci-13-00866-s001.zip › vetsci-4501172-supplementary.pdf]

Supplementary Materials

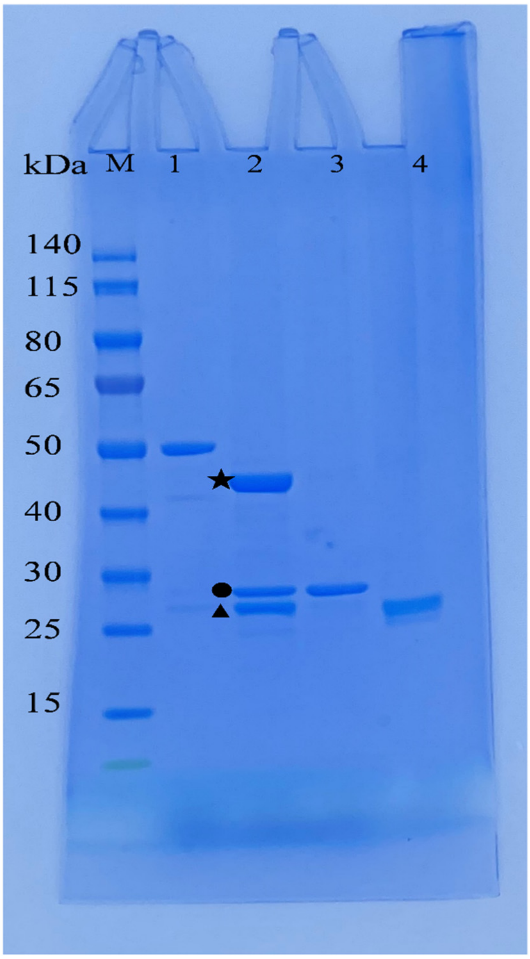

Figure S1. SDS-PAGE analysis of purified and cleaved proteins. Lane M: protein marker; Lane 1: purified GST-EQ8–SclF fusion protein (~50 kDa); Lane 2: cleavage mixture after PreScission protease digestion (★: GST-tagged PreScission protease at ~44 kDa; ●: untagged EQ8–SclF at ~28–30 kDa; ▲: released GST tag at ~26 kDa); Lane 3: purified untagged EQ8–SclF protein after secondary purification (~28–30 kDa); Lane 4: purified GST tag protein (~26 kDa).

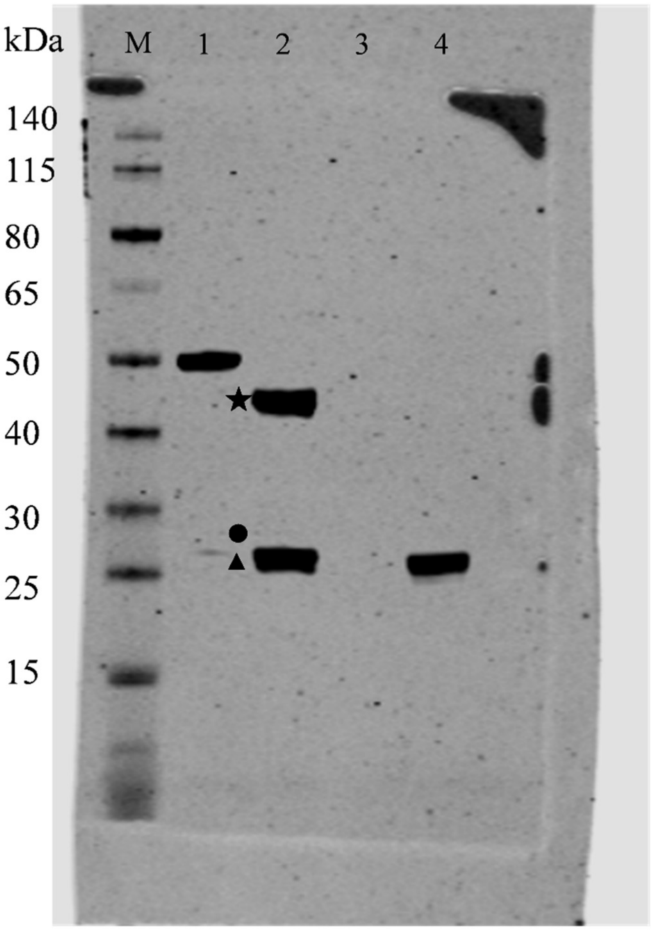

Figure S2. Western blot probed with strangles-positive equine serum. Lane M: protein marker; Lane 1: purified GST-EQ8–SclF fusion protein (~50 kDa); Lane 2: cleavage mixture after PreScission protease digestion (★: GST-tagged PreScission protease at ~44 kDa; ●: untagged EQ8–SclF at ~28–30 kDa; ▲: released GST tag at ~26 kDa); Lane 3: purified untagged EQ8–SclF protein after secondary purification (~28–30 kDa); Lane 4: purified GST tag protein (~26 kDa). The results confirmed that the untagged EQ8–SclF protein retained strong immunoreactivity.

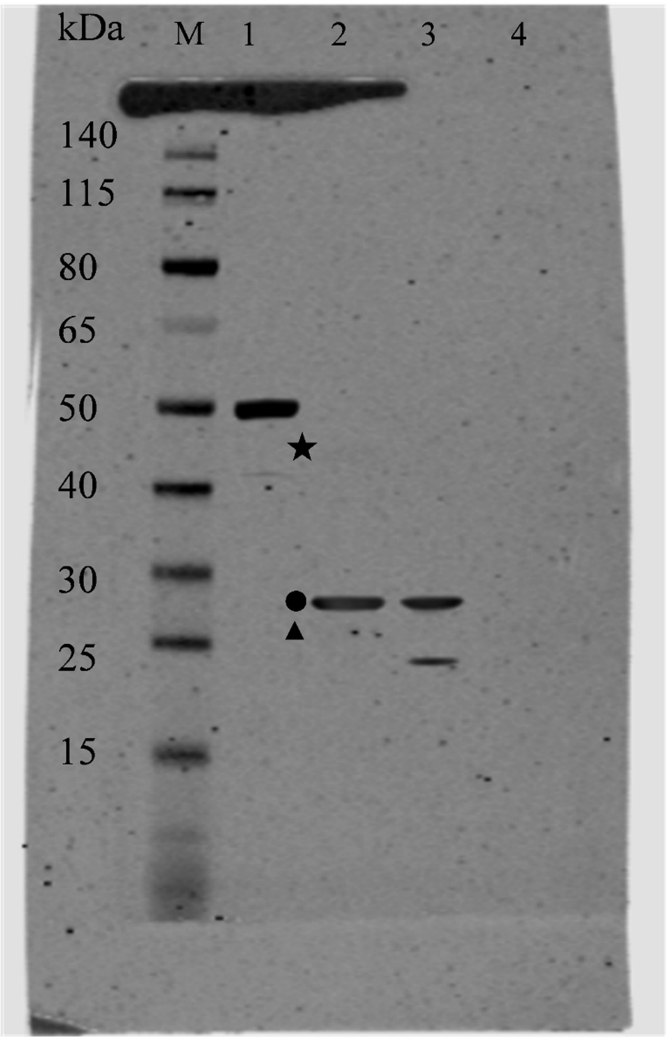

Figure S3. Western blot probed with anti-GST antibody. Lane M: protein marker; Lane 1: purified GST-EQ8–SclF fusion protein (~50 kDa); Lane 2: cleavage mixture after PreScission protease digestion (★: GST-tagged PreScission protease at ~44 kDa; ●: untagged EQ8–SclF at ~28–30 kDa; ▲: released GST tag at ~26 kDa); Lane 3: purified untagged EQ8–SclF protein after secondary purification (~28–30 kDa); Lane 4: purified GST tag protein (~26 kDa). The results confirmed that the final purified EQ8–SclF protein was free of GST tag contamination.

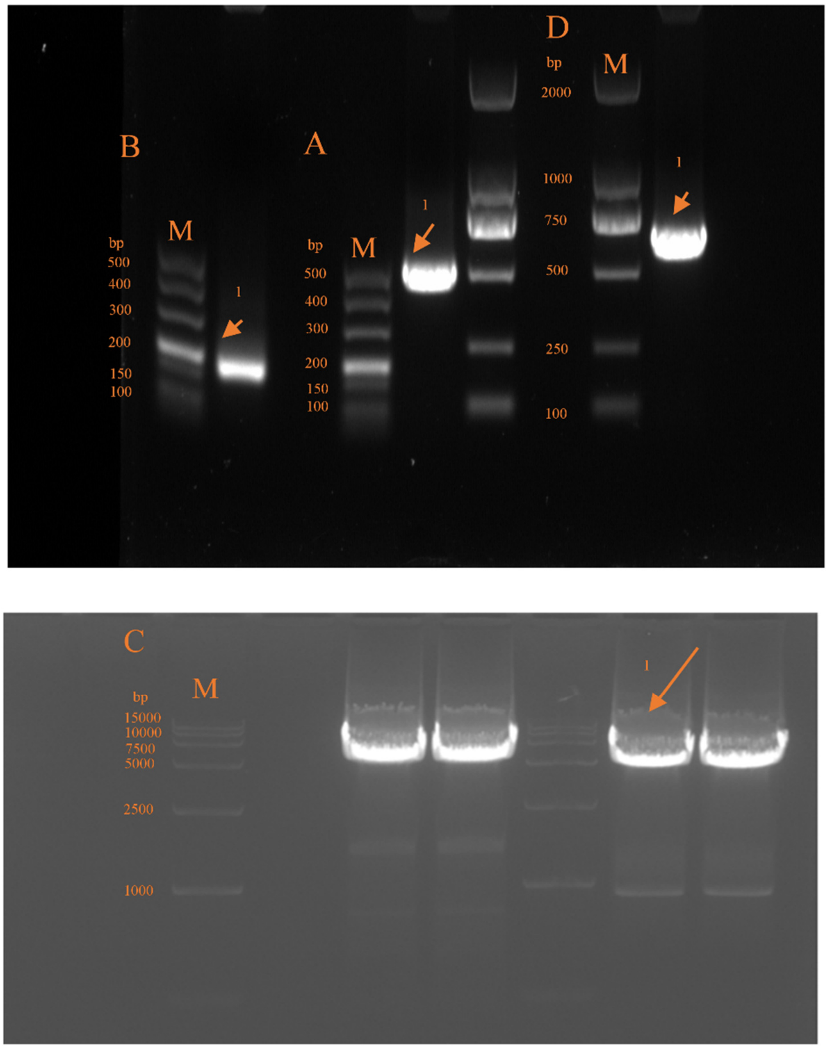

Figure S4. PCR amplification and restriction digestion verification of the recombinant plasmid pGEX-6p-1-EQ8–SclF. (A) PCR amplification of the EQ8 fragment. Lane M: DL500 DNA marker; Lane 1: EQ8 amplicon (450 bp). (B) PCR amplification of the SclF fragment. Lane M: DL500 DNA marker; Lane 1: SclF amplicon (132 bp). (C) Linearized pGEX-6p-1 vector fragment. Lane M: DL15000 DNA marker; Lane 1: linearized pGEX-6p-1 vector (4984 bp). (D) Double digestion verification of the recombinant plasmid with *BamH* I and *XhoI*. Lane M: DL2000 DNA marker; Lane 1: released EQ8–SclF insert fragment (618 bp). The expected sizes of the amplified and digested fragments were consistent with the design, confirming successful construction of the recombinant plasmid.

Supplementary Table S1. Complete data for inter-assay reproducibility of the indirect ELISA. Twenty-four serum samples covering different antibody levels (negative, weakly positive, and strongly positive) were tested on two independently coated ELISA plates, with each sample run in duplicate. OD_{450}-1 and OD_{450}-2 represent the absorbance values obtained from the two independent plates. CV (%) was calculated as SD/mean × 100%.

Sample No.	OD_{450}-1	OD_{450}-2	CV (%)
1	0.077	0.083	5.30
2	0.079	0.085	5.17
3	0.092	0.084	7.07
4	0.091	0.087	3.16
5	0.084	0.082	1.69
6	0.103	0.092	7.78
7	0.092	0.086	4.72
8	0.099	0.094	3.63
9	0.213	0.226	4.18
10	0.095	0.092	2.24
11	0.089	0.092	2.31
12	0.091	0.092	0.78
13	0.089	0.079	8.33
14	0.084	0.083	0.84
15	0.080	0.085	4.22
16	0.109	0.098	7.48
17	0.105	0.092	9.29
18	1.856	1.906	1.88
19	1.687	1.621	2.82
20	3.268	3.316	1.03
21	1.647	1.634	0.18
22	2.001	2.011	0.35
23	2.888	2.890	0.05
24	1.979	1.836	5.30

Note: Twenty-four serum samples covering different antibody levels (negative, weakly positive, and strongly positive) were tested on two independently coated ELISA plates, with each sample run in duplicate. OD_{450}-1 and OD_{450}-2 represent the absorbance values obtained from the two independent plates. CV (%) was calculated as SD/mean × 100%.
